# Estimation of genetic parameters and genome-wide association study for carcass traits in native chickens

**DOI:** 10.5713/ab.25.0070

**Published:** 2025-04-04

**Authors:** Xianghua Zhu, Houxue Cui, Nanxi Dong, Lu Liu

**Affiliations:** 1 Key Laboratory of Applied Technology on Green-Eco-Healthy Animal Husbandry of Zhejiang Province, College of Animal Science and Technology & College of Veterinary Medicine, Zhejiang A&F University, Hangzhou, China; 2 Jinhua Jinfan Feed Co., Ltd, Jinhua, China; 3 College of Animal Sciences, Zhejiang University, Hangzhou, China

**Keywords:** Breeding, Carcass Traits, Chicken, Genome-Wide Association Study, IGF2BP1

## Abstract

**Objective:**

Global consumption for chicken meat is steadily increasing. Carcass traits (e.g., slaughter weight [SW], eviscerated weight [EW]) are important economic traits for the meat production in chickens. Detection of genetic variants for these traits contributes to elucidating the genetic mechanism and accelerating genetic improvement.

**Methods:**

In this study, we performed a genome-wide association study on SW, EW, and thigh muscle weight (ThW), as well as the relative weight of these traits in 565 Sanhuang (SH) chickens. Additionally, genetic estimation has been conducted based on whole-genome variants for carcass traits. Finally, we compared the expression abundance of candidate genes to validate the function on carcass traits.

**Results:**

Except the percentage of SW (SP, 0.08), other traits were detected with moderate to high heritability (0.20−0.50). A high genetic correlation (0.72−0.93) was found among the carcass traits (SW, EW, and ThW). A total of 311 single-nucleotide polymorphisms and 73 candidate genes (e.g., *IGF2BP1*, *BMP3*, *ACSL5*) were identified significant association with carcass traits. *IGF2BP1* was detected a causal role in EW and ThW, *GIP*, *SNF8*, and *PHOSPHO1* were also located within the same genomic peak. *CCND2* was related to SP and significantly expressed in commercial broilers and SH chickens. Lipid metabolism and immune function were simultaneously altered as a result of the selection for carcass traits. The genes *ACSL5* and *RASGEF1B* were also significantly up-regulated in breast muscle in commercial broilers compared to SH chickens. Additionally, the transcriptomic profile of 73 candidate genes was constructed in multi-tissues, and a total of 17 genes (e.g., *IGF2BP1*, *RASGEF1B*, *BMP3*) were defined as differentially expressed genes between commercial broilers and SH chickens.

**Conclusion:**

In general, the findings in this study could facilitate the understandings of genetic mechanisms of carcass traits in chickens, and provide important variants and genes set for genomic breeding.

## INTRODUCTION

The increasing proportion of energy contributed by animal foods in daily diet benefits from the improvement in standards of living as well as an increase in awareness of meat nutritive values [1]. Global demand for meat has increased by 70 million tons in 1961 and 356 million tons in 2022. Meat contains about 20% protein and is abundant in essential micronutrients (zinc, iron, selenium, B vitamins, etc.), which make it as an important food for human nutrition [2]. A moderate increase in meat consumption will certainly improve the nutritional adequacy of diets and health outcomes. While several large-scale epidemiological studies have showed that a high consumption of red and especially processed meat with a higher risk of developing various chronic diseases [3]. Poultry meat, high in protein and low in fat, has not been shown to be associated with the above diseases [4]. Nowadays poultry meat consumption is steadily increasing worldwide.

Among poultry meat products, chicken carcasses, cuts, and processed products are the most consumed (~75% of total poultry meat) followed by turkey (~25%) and, to a lesser extent, duck [5]. The heritability of chicken carcass traits was moderate to high and differed between populations and traits, for example, the estimations of heritability for body weight, breast muscle weight, abdominal fat weight ranged from 0.17 to 0.34 [6]. Using genetic markers associated with the genes involved in carcass traits, genetic improvement could be achieved efficiently on these traits. Genome-wide association studies (GWAS) allow the identification of single-nucleotide polymorphisms (SNPs) associated with effective genes that influence these traits, providing a better biological understanding of the trait and a list of candidate genes for fine mapping [7], especially for chickens, the causal genes related to breast muscle, meat flavor, morphological traits, etc. have been revealed [8–10]. However, these causal genes may not be effective in all populations due to differences in selection backgrounds and demographic histories among different chicken breeds. Therefore, under the premise of existing reference genes, it is necessary to perform GWAS for carcass traits in specific chicken populations.

Currently, partial genes have been proved to be effective for carcass traits. For example, the IGF2BP1 gene plays an important role in regulating body size and carcass traits in both duck and chicken [11–13]. After long-term domestication and selection, the genetic variants regulating IGF2BP1 transcription were usually fixed and conserved in commercial chickens, while it’s dispersed in various native chickens. In this study, we aimed to detect genomic peaks and candidate genes associated with carcass traits based on whole-genome sequencing in Sanhuang (SH) chickens. Simultaneously, we compared the expression level of candidate genes in multiple tissues and chicken breeds to refine the functional genes profile, which provided novel insights into genetic mechanism of production traits and facilitated the genomic breeding in the view of biomarker selection associated with carcass traits.

## MATERIALS AND METHODS

All procedures followed the guidelines of the Institutional Animal Care and Use Committee at Zhejiang A&F University, China (No. ZAFUAC202434).

### Experimental chickens and phenotypic measurement

A total of 565 SH chickens were used in this study and all birds were raised in three-stair step cages (one bird per cage) under the same recommended environmental and nutritional conditions. Basal diets (containing 15.2% crude protein and 18.3 MJ/kg gross energy) were formulated based on the National Resource Council requirements, feed and water were provided *ad libitum* during this study. Blood samples were obtained using standard venipuncture techniques at 91 days of age. Anticoagulated blood samples were stored at −20°C for genomic DNA extraction. After blood collection, all chickens were euthanized under carbon dioxide anesthesia by severing the carotid artery. After slaughter, slaughter weight (SW), eviscerated weight (EW), and thigh muscle weight (ThW) of each chicken were weighted and recorded. The percentage of these three traits (percentage of slaughter weight [SP], percentage of eviscerated weight; [EP], percentage of thigh muscle weight [ThP]) were calculated according to the standard procedures.

### DNA extraction and whole-genome sequencing

DNA was extracted from blood samples using the phenol-chloroform method. A total of 565 DNA samples were used for whole-genome resequencing. The quality and quantity of DNA were examined using a NanoDrop device and by agarose gel electrophoresis. Then, paired-end libraries were generated for each eligible sample using standard procedures. All libraries were sequenced on an Illumina HiSeq X Ten platform and each individual at less with over 10 G raw read sequence.

### Genotyping, quality control, and imputation

Filtered FASTQ data (parameters: −q 30; −u 30; −l 150) (https://github.com/OpenGene/fastp) were aligned to the reference genome [Gallus_gallus-6.0 (GCA_000002315.5)] using BWA MEM (v 0.7.10) with default parameters [14]. Mapped reads were converted into BAM files using SAMtools (v 0.1.18), and duplicate reads were removed by SAMtools and Picard MarkDuplicates (http://broadinstitute.github.io/picard). After mapping, SNP calling was performed using exclusively GATK (v 3.5), and the output was further filtered using VCFtools (v 0.1.15) [15,16]. Genotype quality control was conducted using PLINK (v 1.90) [17] to detect and exclude unreliable genotypes. SNPs were selected based on SNP maximum missing rate <5%, minor allele frequency >0.05. Moreover, individuals were excluded due to sample call rate <95%. Totally, 9402908 SNPs on autosomes were retained and missing alleles were imputed using Beagle 5.2 [18].

### Genome-wide association study for carcass traits

To minimize false positives, it is essential to account for population structure and family effects. Mixed linear model (MLM) is an effective approach to address population stratification in GWAS. An efficient MLM was used for the association analysis using the GEMMA software [19]. SNP effect was set as fixed effect and kinship matrix as random effect. The GWAS was performed for carcass traits as follows:


y=Wα+xβ+μ+ɛ,

where y indicates the phenotypic record of carcass traits; W indicates the covariates matrix, including the top three principal component analysis value; α is vector of the corresponding coefficient (including the intercept); βis the SNP effect; μ~MVN_n_ (0, λτ^−1^K), μ indicates random polygenic effect, ε is the residual error.

The threshold of genome-wide significance was assessed by simpleM and the Bonferroni correction methods [20]. The significance threshold was 0.05/339733 (−log10 *p* = 6.83) and the suggestive significance threshold was 1/339733 (−log10 p = 5.53). In this study, SNPs that reached the suggestive significance threshold were identified as significant SNPs associated with traits. Within the peak, the SNP information and candidate genes were presented.

### Linkage disequilibrium analysis

The Linkage disequilibrium (LD) between the peak SNP and surrounding SNPs within the target regions was calculated using PLINK v1.9 [17], and the haplotypes were genotyped based on candidate SNPs and visualized using LDBlockShow software (v 4.3) [21].

### Estimation of heritability

Genomic SNPs information were used to generate genomic relationship matrix (GRM) by the approach from VanRaden [22], and variance component estimates for traits were calculated based on univariate animal model constructed by ASReml v4.1. The univariate model was defined as follows:


y=Xb+Za+e,

where y is the vector of phenotype, b is the vector of fixed effects, including batch effect, a is the vector of random additive genetic effects, e is the vector of random residual effect. X and Z are design matrices relating observation to the corresponding fixed and random effects, respectively. The variance-covariance structure assumed for random effect was following:


$$\text{var}\left[\begin{array}{c} a\\ e \end{array}\right]=\left[\begin{array}{cc} G\sigma_{a}^{2} & 0\\ 0 & I{\mathrm{s\sigma}}_{e}^{2} \end{array}\right],$$

where G is the GRM based on SNP data, I is the identity matrix, σa^2^ is the additive genetic variance, σe^2^ is the residual variance. The heritability of phenotypes was calculated by: h^2^ = σa^2^/(σa^2^ + σe^2^).

### Estimation of genetic and phenotypic correlation

A bivariate animal model was used to evaluate the genetic correlation and phenotypic correlation between any two traits:


$$\left[\begin{array}{c} {\text{y}}_{\text{1}}\\ {\text{y}}_{\text{2}} \end{array}\right]=\left[\begin{array}{cc} {\text{X}}_{1} & 0\\ 0 & {\text{X}}_{2} \end{array}\right]\left[\begin{array}{c} {\text{b}}_{\text{1}}\\ {\text{b}}_{\text{2}} \end{array}\right]+\left[\begin{array}{cc} {\text{Z}}_{1} & 0\\ 0 & {\text{Z}}_{2} \end{array}\right]\left[\begin{array}{c} {\text{a}}_{\text{1}}\\ {\text{a}}_{\text{2}} \end{array}\right]+\left[\begin{array}{c} {\text{e}}_{\text{1}}\\ {\text{e}}_{\text{2}} \end{array}\right],$$

where y, a, b, e, X, and Z were same as above equation.

### RNA extraction and mRNA sequencing

Total RNA was extracted from breast muscle, thigh muscle, lung, liver, heart, and fat (n = 5) tissues in SH chickens of 91 d using TRIzol reagent (15596026CN; Thermo Fisher Scientific, Waltham, MA, USA). And breast muscle from commercial broilers and SH chickens (n = 6) of 40 d was also conducted RNA isolation. A total of 42 RNA samples were used to mRNA library construction and sequencing based on Illumina platform (PE150), and >6 G raw reads per sample were produced.

### Transcriptomic analysis

The bioinformatic pipeline of mRNA sequencing followed the methodology outlined in previous report [10]. Briefly, the raw reads were firstly trimmed by Fastp (v0.21) software (−q 20 −u 30 −n 5 −l 150) [23], and qualified reads were used to genome alignment (Gallus_gallus-6.0 [GCA_000002315.5]) using HISAT2 (v2.2.0) software [24] with default parameters. After data conversion, quality control, and index, transcript assembling and quantification were implemented by StringTie (v2.1.6) software (−l 150) [25]. Differentially expressed genes were defined when fold change > 1.5 or < 0.67 and p<0.05 using DESeq2 software [26].

### Real-time polymerase chain reaction for candidate genes

Gene expression of partial candidate genes (e.g., *IGF2BP1*, *TBXAS1*, *SKAP1*) for carcass traits were examined with real-time polymerase chain reaction (RT-PCR). Total RNA from breast muscle of commercial broilers and SH chicken (n = 6) was isolated using TRIzol reagent (15596026CN, Thermo Fisher Scientific). The measurement of RNA concentration and integrity was conducted with Agilent 2100 Nano and gel electrophoresis. Next, the total RNA was reverse transcribed to cDNA using FastKing RT kit (KR116; TIANGEN, Beijing, China). And the reaction system of RT-PCR was created using SYBR Green Pro Taq HS kit (AG11740; Accurate Biology, Changsha, China). The specific primers were designed based on Oligo 7.0 software (Supplement 1). The condition detail was as follows: 94°C for 10 min, followed by 35 cycles of 94°C for 3 s and an annealing temperature of 32 s. β-actin was regarded as the internal controls. The expression level was compared using the 2^−ΔΔCT^ method.

### Statistical analysis

Significance of expression differences between commercial broilers and SH chickens was tested by the Student t-test, and the phenotypic alteration (e.g., EW, SW, ThW) among different chickens with various haplotypes was conducted by Kruskal-Wallis test using the SPSS Version 22.0 (IBM, Armonk, NY, USA). Confidence limits were set at 95% and p<0.05 (*) or p<0.01 (**) was considered significant. Data are presented as the mean±standard deviation.

## RESULTS

### Descriptive statistics and heritability estimation

**Figure 1:**
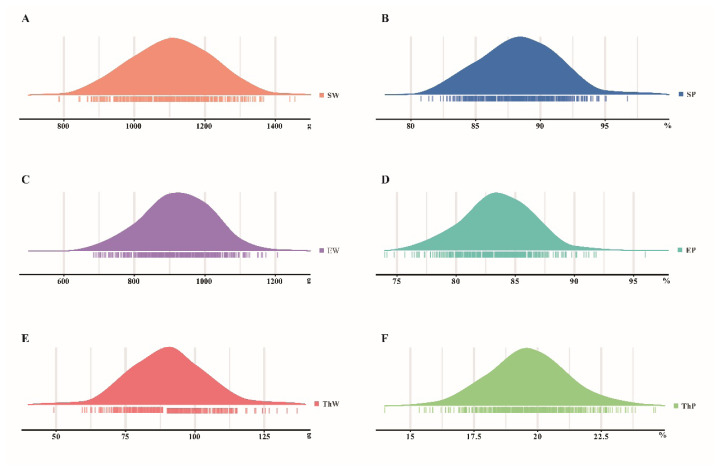
Distribution results of six carcass traits. SW, slaughter weight; EW, eviscerated weight; ThW, thigh muscle weight; SP, percentage of slaughter weight; EP, percentage of eviscerated weight; ThP, percentage of thigh muscle weight.

Descriptive statistics of the carcass traits (SW, SP, EW, EP, ThW and ThP) were presented in Table 1. The average SW, EW, and ThW in SH population was 1,107.97 g, 923.12 g, and 181.50 g, respectively, the average SP and EP was more than 88% and 73%, respectively, and the ThP ranged from 11.94% to 24.64%. The coefficients of variation (CV) of relative weight (SP, EP, ThP) were maintained below 10%, while the CVs of SW, EW, and ThW were relatively high (>10%). All these traits were followed a normal distribution (Figure 1).

### Genetic and phenotypic correlations among carcass traits

Estimates of heritability, genetic and phenotypic correlation based on genomic information were shown in Table 2. The estimates of heritability of six traits were calculated and ranged from 0.08 to 0.50, five of them (besides SP) were classified as traits with moderate to high heritability. High estimates of genetic correlation among SW, EW, and ThW were obtained (ranged from 0.83 to 0.95), and higher results were also found between SW and SP, ThW and ThP, EW and EP. As expected, similar results of phenotypic correlation among these traits were detected. High phenotypic correlation among SW, EW, and ThW was presented (0.72 to 0.93), a moderate to high phenotypic correlation between SW and SP, EW and EP, ThW and ThP was obtained. Additionally, there was a relative low correlations among SP, EP, and ThP.

### Genome-wide association studies for slaughter weight and percentage of slaughter weigh

**Figure 2:**
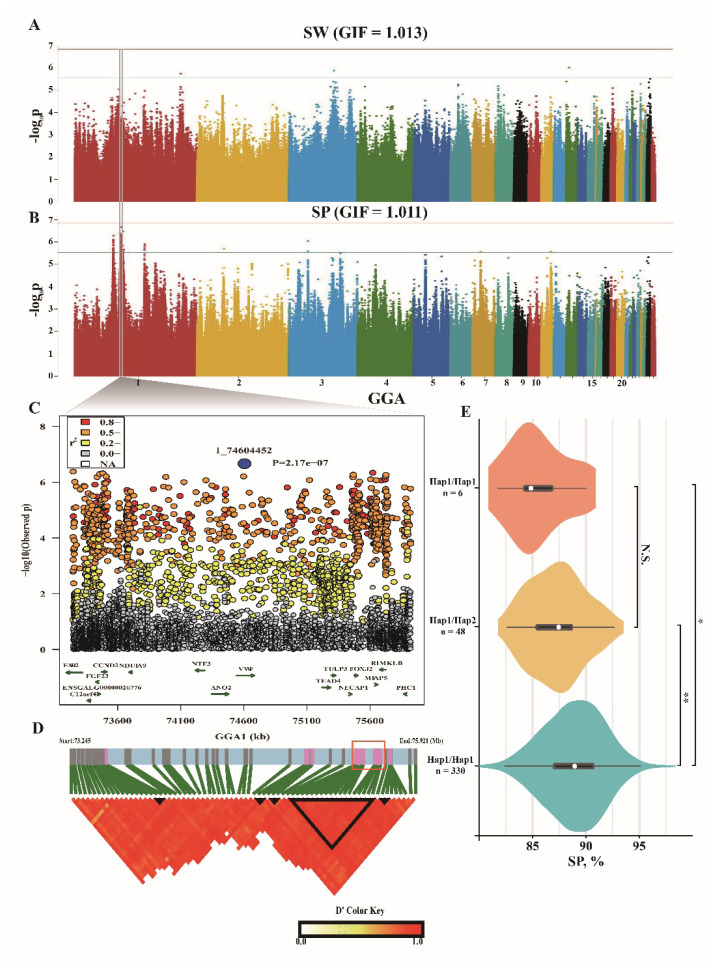
GWAS results for SW and SP traits. (A,B) Manhattan plot for SW and SP based on GWAS, the genome inflation factors were provided. (C) Fine mapping of SP trait on GGA1, only the genes covered by significant SNPs were annotated and presented. (D) LD block result based on significant SNPs. (E) Effect of candidate haplotype on SP trait. * p<0.05, ** p<0.01, SW, slaughter weight; SP, percentage of slaughter weight; N.S., no significance; GWAS, genome-wide association studies; LD, linkage disequilibrium; SNPs, single-nucleotide polymorphisms.

For SW trait, only 3 associated SNPs were detected in GGA1, GGA3, and GGA13 (Figure 2A). The SNP on GGA1 was calculated the high SNP effect (−65.54) and low allele frequency (0.06), while the SNP on GGA13 was identified with a high phenotypic variance explained (PVE) (4.94%) (Table 3). These 3 SNPs were located within *RNASEH2B*, *MMS22L*, *SPDL1*, and *DOCK2*. For SP trait, a total of 8 significant peaks were found on GGA1, GGA2, GGA3, GGA7, and GGA11 (Figures 2B, 2C). Four of which were located on GGA1, and only one intergenic SNP was identified on GGA2 and GGA7, respectively. Among these SNPs, phenotypes can be explained up to 5.59% by the lead SNP (Table 3), and one major haplotype covering 18 SNPs was formed within the lead peak (Figure 2D), the major haplotype was found a significant enhancing effect on SP (Figure 2E). In different peaks, the genes *TEAD4*, *FOXJ2*, *CCND2*, *ZNF384*, and others were covered by significant SNPs.

### GWASGenome-wide association studies for eviscerated weight and percentage of eviscerated weight

**Figure 3:**
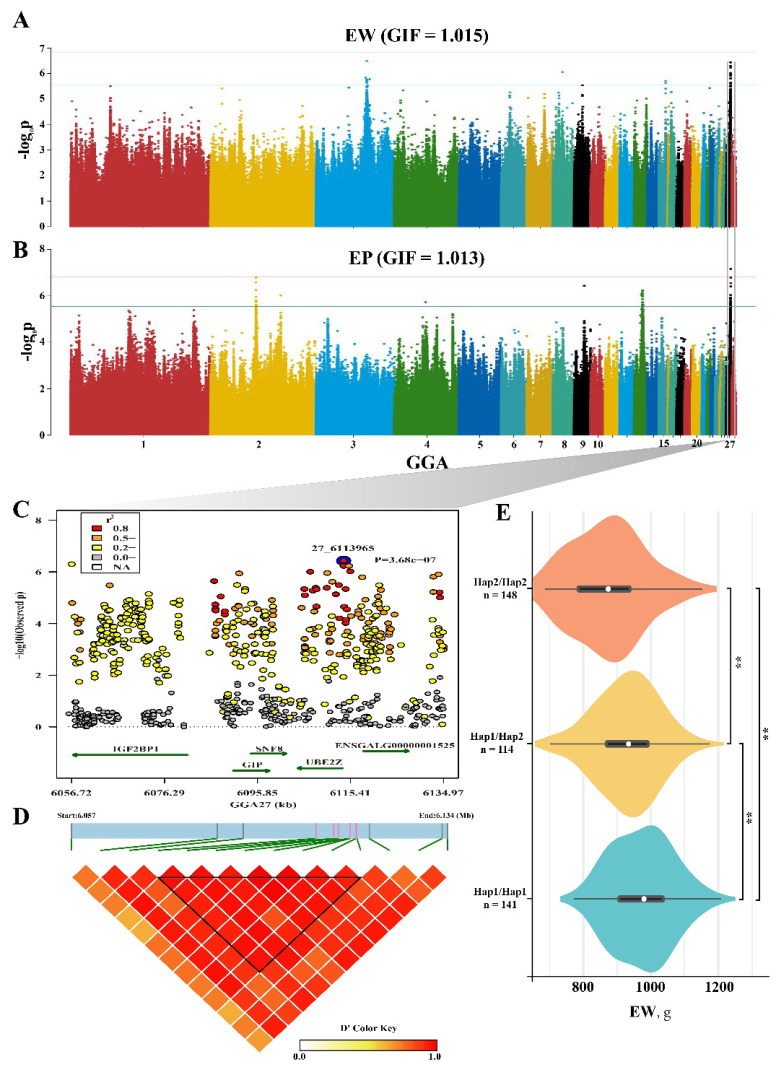
GWAS results for EW and EP traits. (A,B) Manhattan plot for EW and EP based on GWAS, the genome inflation factors were provided. (C) Fine mapping of EW trait on GGA27, only the genes covered by significant SNPs were annotated and presented. (D) LD block result based on significant SNPs. (E) Effect of candidate haplotype on EW trait. ** p<0.01. EW, eviscerated weight; EP, percentage of eviscerated weight; GWAS, genome-wide association studies; SNPs, single-nucleotide polymorphisms; LD, linkage disequilibrium.

GWAS peaks for EW and EP were presented in Figures 3A, 3B. For EW trait, we found 5 peaks (GGA3, GGA8, GGA9, GGA15, and GGA27) were correlated to EW (Figure 3A). On GGA3, six significant SNPs spanned 5 Mb contributed more than 4.7% of phenotypic variation, and this peak included candidate genes *USP45*, *PNISR*, *MMS22L*, etc. (Table 4). On GGA27, a ~80 kb genomic interval covered 14 SNPs was found significant associations (Figure 3C), and an obvious haplotype including 8 SNPs was detected additive effect on EW (Figures 3D, 3E). *IGF2BP1* gene, related to growth traits in farm animals, was annotated by the candidate SNPs. And *GIP*, *SNF8*, and others were also included in this peak. Additionally, only 1 to 2 associated SNPs located on GGA8, GGA9, and GGA15. For the EP trait, three prominent peaks were identified on GGA2, GGA13, and GGA27. Additionally, *IGF2BP1* showed an association with EP (Supplement 2), and a haplotype located in the upstream of *IGF2BP1* exhibited a significant additive correlation with EP (Supplements 2, 3). And the genes *BMP6*, *PTPRM*, *SGCD*, etc. on GGA2 and GGA13 were also correlated to EP. Additionally, single SNP on GGA4 and GGA9 was annotated intergenic variant and contributed more than 4.8% of PVE to EP (Table 4).

### Genome-wide association studies for thigh muscle weight and percentage of thigh muscle weight

**Figure 4:**
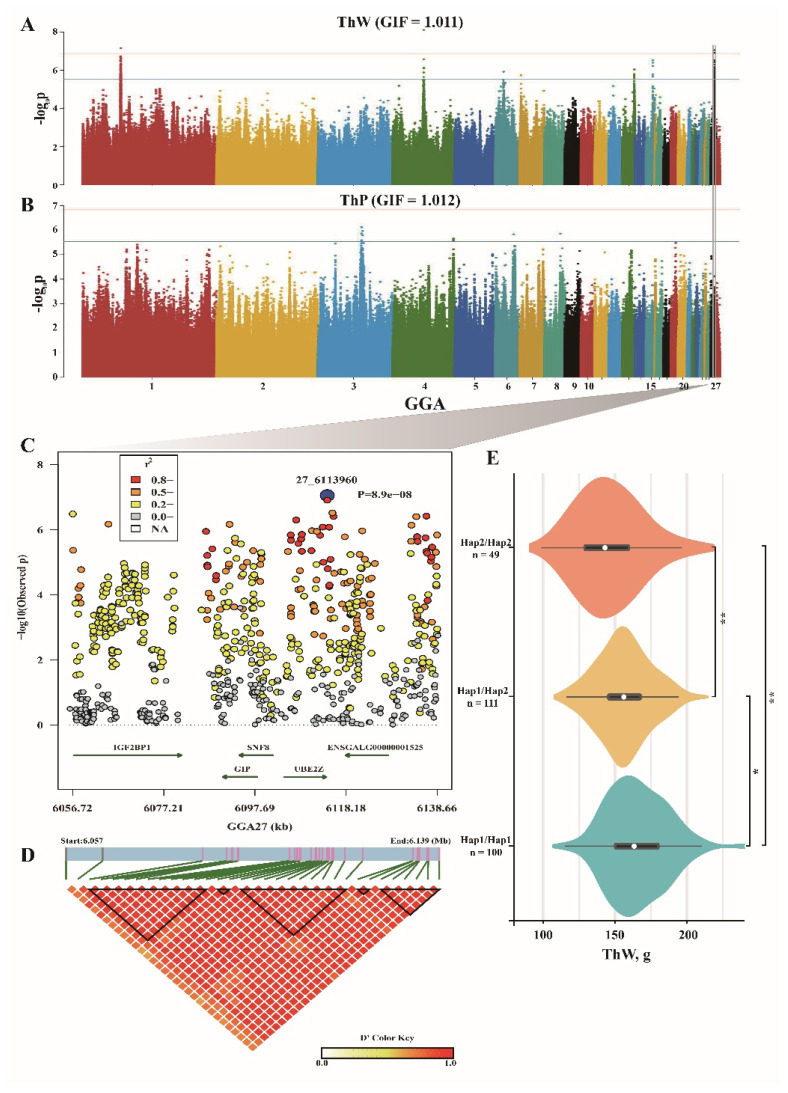
GWAS results for ThW and ThP traits. (A,B) Manhattan plot for ThW and ThP based on GWAS, the genome inflation factors were provided. (C) Fine mapping of ThW trait on GGA27, only the genes covered by significant SNPs were annotated and presented. (D) LD block result based on significant SNPs. (E) Effect of candidate haplotype on ThW trait. * p<0.05, ** p<0.01. ThW, thigh muscle weight; ThP, percentage of thigh muscle weight; GWAS, genome-wide association studies; SNPs, single-nucleotide polymorphisms; LD, linkage disequilibrium.

GWAS peaks for ThW and ThP were presented in Figures 4A, 4B. For ThW trait, five peaks and two isolated SNPs on different chromosomes were identified (Figure 4A). Similar to EW and EP, a peak spanned ~90 kb on GGA27 (including 33 SNPs) was highly correlated to ThW, and *IGF2BP1*, *GIP*, *SNF8*, etc. were covered by these SNPs (Figure 4C). Additionally, three haplotypes were also found important association with ThW (Figures 4D, 4E, Supplements 5, 6). On GGA1, the region from 55,911,047 to 56,322,618, including 71 SNPs, contributed more than 4.5% of PVE to ThW (Supplement 7; Table 5), three haplotypes were revealed in this peak, but a rare haplotype frequency related to enhancing ThW was detected (Supplements 8–11). By annotation, *TBXAS1*, *HIPK2*, and *ENSGALG00000012796* were located within this region (Supplement 7). On GGA4, peak SNP was detected and contributed 6.85% of PVE to ThW, a rare haplotype was identified within this region and *BMP3* gene was found (Supplements 12–14). Additionally, a short peak covering less than 10 SNPs were detected on GGA6 (1), GGA7 (1), GGA13 (7), and GGA15 (6), the genes *ARHGAP26*, *CIT*, *NR3C1*, etc. were overlapped by these candidate regions (Table 5). For ThP, two associations on GGA3 and GGA4 were obtained based on the threshold (Figure 4B), only *ENSGALG00000015017* and *ATRN* genes were annotated. On GGA6 and GGA8, single SNPs were found a significant correlation to ThP, and *BEND5* gene was annotated and putatively related to thigh muscle trait (Table 5).

### Expression of candidate genes for carcass traits

**Figure 5:**
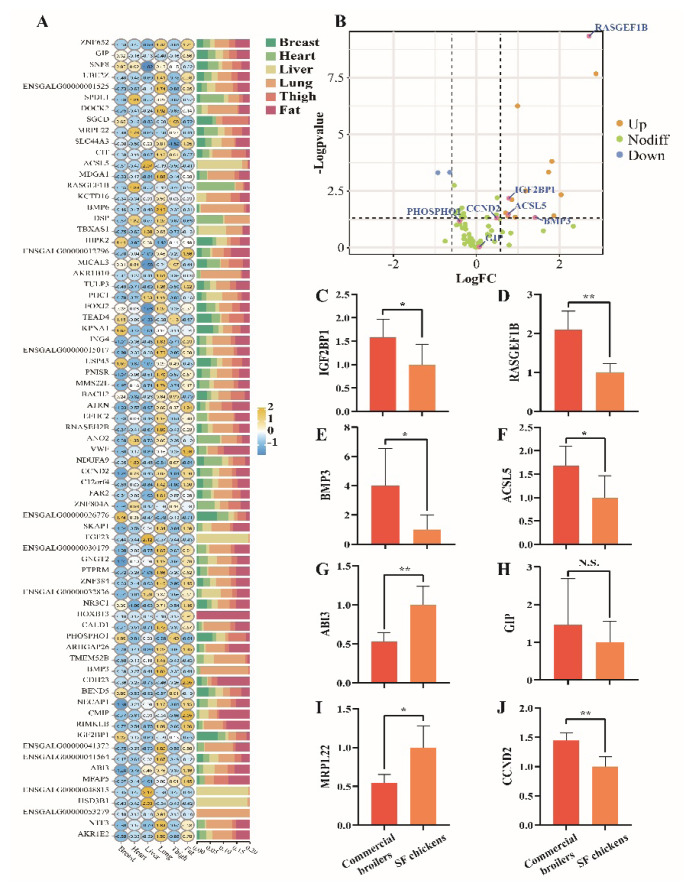
Expression profile of candidate genes. (A) Expression profile of 73 candidate genes in breast muscle, heart, liver, lung, thigh, and fat tissues. (B) Identification of DEGs for 73 candidate genes. Blue dots indicate down-regulated genes, orange dots indicate up-regulated genes, green dots indicate genes detected no difference, and pink dots indicate selected candidate genes related to carcass traits based on annotation. (C-J) Comparison of gene expression for *IGF2BP1, RASGEF1B, BMP3, ACSL5, ABI3, GIP, MRPL22*, and *CCND2*, respectively. * p<0.05, ** p<0.01, N.S., no significance.

Based on GWAS summary, a total of 73 protein-coding genes were annotated by candidate SNPs (Supplement 15). Firstly, an expression profile of multi-tissues was founded in SH chicken. And *ACSL5*, *HSD3B1*, *BMP3*, etc. were transcribed specifically in liver, lung, and other tissues (Figure 5A). Next, we compared expression of these genes in muscle of commercial broilers and SH chickens. A total of 17 of 73 genes were defined as differentially expressed gene (DEGs), 15 of which were up-regulated in commercial broilers, while *ABI3* and *MRPL22* were down-regulated (Figure 5B). Additionally, cell cycle related gene *CCND2* was found a trend of increased expression (Fold change = 1.41, p = 0.051). To ascertain the accuracy of sequencing results, we performed quantitative polymerase chain reaction assay for partial genes. Similarly, *IGF2BP1*, *BMP3*, *ACSL5*, and *RASGEF1B* were highly transcribed in commercial broilers, while *MRPL22* and *ABI3* were restricted, no difference was detected between two chicken breeds for muscular *GIP* (Figures 5C–5I). We also confirmed transcriptional difference of *CCND2* between two chicken breeds, and significant enhancement was detected in commercial broilers (Figure 5J). All these results proved the accuracy of sequencing results and conclusion.

## DISCUSSION

GWAS is a powerful tool for the genetic analysis of important production traits in farm animals [9,27]. Carcass traits are important economic traits, which directly affect the meat production and play an important role in chicken industry. Previously study showed that the heritability of these traits in chicken was moderate to high [6]. Similarly, we also detected a high heritability of EW, SW, ThW, etc., except for SP (h^2^ = 0.08). Low selective pressure on SP trait may be the reason for low heritability, and limited sample size and absence of other genomic variants (e.g., structural variants) could also be important reasons. High genetic correlations (>0.7) between SW and SP, ThW and ThP were observed, as well as among the SW, EW, and ThW. Which was highly consistent with previous reports. However, it’s moderate between EW and EP (0.24). Totally, mutations associated with these traits can be obtained from GWAS and applied to genomic breeding to improve the performance of meat production.

Usually, GWAS peaks include the causal variants and adjacent SNPs due to the hitchhiking effect, herein, the LD blocks reduce the possibility of causal SNPs and resulted in a high false positive rate in GWAS. In current study, we obtained the effective calculations using simpleM method to avoid the bias from LD, and controlled the false positive rate by Bonferroni correction. Based on that, we found that the significant peaks related SW, EW, and ThW were not completely identical to the SP, EP, and ThP. In an unselected population, the different peak and low significance were often detected for related carcass traits [28], consistent associations with different carcass traits could be revealed in an F2, for example, the effect of terminal region of GGA4 and GGA27 on carcass traits (e.g., body weight, shank length, EW) [29].

For farm animals, carcass traits are typical complex phenotypes, which highly correlated to the muscle development. Genomic peaks on GGA27 have been found significant associations with carcass and growth traits [12,13,29], candidate genes *IGF2BP1*, *GIP*, *PHOSPHO1*, etc. were reported to be correlated to skeletal muscle (breast and thigh muscle) development. Especially for *IGF2BP1*, the causal role in body size and carcass traits has been proved in chickens and ducks, 15% of body size can be enhanced by regulating this gene. *GIP* gene is necessary for bone remodeling and bone quality, and activates osteoblasts and promotes bone development [30]. Similarly, *BMP3* gene is highly correlated with bone and muscle growth [31], both of them contribute substantially to carcass traits. Cell cycle is basic physiological process affecting cell proliferation, differentiation, and repair after muscle injuries. *CCND2* is responsible for the transition of cell cycle from G1 to S phase, and could contribute significantly to the skeletal muscle proliferation and differentiation by subjecting to targeting binding by miR-17 and miR-206 [32,33]. Here, the result that an increasing expression of *CCND2* in breast muscle from commercial broilers was consistent with the production performance.

After long-term selection, carcass traits of commercial broilers significantly outperform that of native chickens, including SH chickens, however, this advantage comes at the price of decreased immunity and a set of undesirable performance [34]; additionally, native chickens provide superior meat flavor compared to commercial broilers. Therefore, growth and carcass traits were closely related to immune and metabolism functions [35], which were characterized by a complicated multi-level molecular regulatory network. Usually, carcass performance was negatively correlated with immune performance and lipid metabolism. *RASGEF1B* gene is involved in immune response during macrophage activation and protection against microbial infections, and could be induced in macrophages on stimulation with toll-like receptor agonists [36,37]. We found *RASGEF1B* was most affected between commercial broilers and SH chickens, supporting the consistency between carcass traits and immune performance. Interestingly, knockdown of circRASGEF1B could alter the cell cycle and mitotic division progression, which may influence the muscle development [36]. Fatty acid component is tightly correlated with the meat flavor and quality. *ACSL5* is a vital factor in fatty acid elongation, degradation, and biosynthesis. Yuan *et al*. have proved that *ACSL5* was associated with meat aldehydes and hexanal components and contributed to flavor formation in native chickens [38]. We have identified the relationship between *ACSL5* and ThP, and *ACSL5* was more enriched in commercial broilers compared to SH chickens. Demonstrating that lipid metabolism in leg had a significant impact on muscle development. On 42 d, commercial broilers were in a period of rapid muscle development with a high energy demand, while SH chickens were in the early developmental stage rather than the fat deposition stage [39]. Therefore, *ACSL5* was more activated in commercial broilers.

In addition to the relationship between genotype to phenotype, we provided a transcriptional profile of candidate genes in part tissues. These evidences refined the range of candidate genes for carcass traits, including *IGF2BP1*, *ACSL5*, *BMP3*, etc., which mainly enriched in muscle growth, metabolism, and immune function. Totally, the findings of this study provide a list of crucial genes (e.g., *IGF2BP1*, *ACSL5*, *BMP3*) related to carcass traits (e.g., SW, EW, ThW) based genomic and transcriptomic evidence. And all these genes could serve as the molecular phenotypes for different carcass traits, and contribute to the development of genomic selection algorithms based on multi-omics technologies [40], which could provide valuable insights into improving carcass traits in chickens.

In SH chickens, the heritability of most carcass traits (except for SP) ranged from moderate to high, and a high genetic correlation was found among carcass traits (except for EW and EP). Based on GWAS summary, a total of 311 SNPs and 73 candidate genes were identified significant associations with six carcass traits (SW, EW, ThW, and relative weight of these traits). And 17 genes (e.g., *IGF2BP1*, *RASGEF1B*, *ACSL5*) were defined as DEGs between commercial broilers and SH chickens. Besides muscle growth and bone formation, the genes involved in lipid metabolism (e.g., *ACSL5*) and immune function (e.g., *RASGEF1B*) were also altered with carcass traits.

## Figures and Tables

**Table 1:** Descriptive statistics of six carcass traits in Sanhuang chickens

Trait	Mean	SD	Quartile 25%	Quartile 75%	Min	Max	CV (%)
SW (g)	1,107.97	114.19	1,027.50	1,182.88	787.00	1,456.50	10.31
SP (%)	88.44	2.76	86.37	90.48	80.81	96.75	3.12
EW (g)	923.12	96.43	858.05	990.50	684.20	1,206.40	10.45
EP (%)	73.70	3.05	81.64	85.09	56.96	85.94	4.14
ThW (g)	181.50	26.06	162.55	197.6	98.60	273.80	14.36
ThP (%)	19.63	1.59	18.64	20.58	11.94	24.64	8.10

SD, standard deviation; CV, coefficients of variation; SW, slaughter weight; SP, percentage of slaughter weight; EW, eviscerated weight; EP, percentage of eviscerated weight; ThW, thigh muscle weight; ThP, percentage of thigh muscle weight.

**Table 2:** Estimation of genetic parameters for carcass traits in Sanhuang chickens^1)^

Trait	SW	SP	EW	EP	ThW	ThP
SW	0.50±0.10	0.95±0.40	0.93±0.01	−0.04±0.24	0.72±0.02	0.25±0.19
SP	0.33±0.04	0.08±0.08	0.24±0.04	0.55±0.03	0.22±0.04	0.10±0.05
EW	0.95±0.02	0.88±0.38	0.41±0.10	0.24±0.24	0.84±0.06	0.20±0.21
EP	0.13±0.05	0.11±0.50	0.39±0.04	0.20±0.10	0.38±0.04	0.17±0.05
ThW	0.83±0.07	0.85±0.42	0.80±0.02	0.14±0.26	0.37±0.10	0.70±0.11
ThP	0.12±0.05	0.21±0.40	0.16±0.05	−0.10±0.30	0.72±0.02	0.31±0.10

1) The upper diagonal is genetic correlation, the lower diagonal is phenotypic correlation, and the diagonal is heritability.

SW, slaughter weight; SP, percentage of slaughter weight; EW, eviscerated weight; EP, percentage of eviscerated weight; ThW, thigh muscle weight; ThP, percentage of thigh muscle weight.

**Table 3:** GWAS summary for SW and SP traits

Traits	Chromosome	Positions (bp)/Region (Mb)	SNP number	MAF	β^1)^	PVE^2)^	Candidate genes
SW	1	171236720	1	0.06	−65.54	4.7	*RNASEH2B*
SW	3	72512309	1	0.45	−33.71	4.83	*MMS22L*
SW	13	4871588	1	0.28	37.39	4.94	*SPDL1, DOCK2*
SP	1	62003190~62517857	20	0.05–0.12	−1.39–−1.98	4.53–5.03	*AKR1E2, CALD1, AKR1B10*
SP	1	73245453–75920649	73	0.07–0.16	−1.12–−1.84	4.53–5.59	*TEAD4, FOXJ2, CCND2*, etc.
SP	1	77173210–79170352	7	0.09–0.12	−1.32–−1.54	4.60–5.38	*TMEM52B, KPNA1, ZNF384*, etc.
SP	1	112416162–112834092	6	0.06–0.33	−0.86–−1.96	4.72–4.96	*EFHC2, ENSGALG00000032836*
SP	2	43997045	1	0.45	−1.84	4.67	\
SP	3	30168222–30207334	2	0.22–0.24	−1.04–−1.07	4.56–5.00	*MDGA1*
SP	7	12533171	1	0.21	−1.06	4.67	\
SP	11	15536095–15641028	2	0.11–0.42	−0.90–−1.42	4.56–4.58	*CMIP*

1) β indicates allele effect calculated from GWAS result.

2) PVE indicates the proportion of phenotypic variance explained by SNPs.

GWAS, genome-wide association studies; SNPs, single-nucleotide polymorphisms; SW, slaughter weight; SP, percentage of slaughter weight; MAF, minor allele frequency.

**Table 4:** GWAS summary for EW and EP traits

Traits	Chromosome	Positions (bp)/region (Mb)	SNP number	MAF	β^1)^	PVE^2)^	Candidate genes
EW	3	70036940–75353475	6	0.14–0.22	32.17–45.54	4.70–5.43	*USP45, PNISR, MMS22L*, etc.
EW	8	13866456	1	0.23	−34.23	4.99	*SLC44A3*
EW	9	12311124	1	0.15	39.02	4.67	\
EW	15	9919244–9919247	2	0.38	−29.29–−29.61	4.63–4.70	*CIT*
EW	27	6056724–6133951	14	0.38–0.50	−27.59–−30.18	4.80–5.40	*IGF2BP1, GIP, SNF8*, etc.
EP	2	64465215–64573464	15	0.44–0.49	−0.83–0.95	4.56–5.69	*BMP6, DSP*
EP	2	99365373	1	0.33	−0.9	5.03	*PTPRM*
EP	4	43832794	1	0.36	−0.89	4.81	\
EP	9	14985129	1	0.14	−1.3	5.34	\
EP	13	11155184–13283919	14	0.25–0.47	0.82–1.02	4.59–5.29	*SGCD, MRPL22*
EP	27	6003735–6359668	22	0.16–0.40	−0.93–−1.20	4.58–6.00	*PHOSPHO1, IGF2BP1, GIP*, etc.

1) β indicates allele effect calculated from GWAS result.

2) PVE indicates the proportion of phenotypic variance explained by SNPs.

GWAS, genome-wide association studies; SNPs, single-nucleotide polymorphisms; EW, eviscerated weight; EP, percentage of eviscerated weight; MAF, minor allele frequency.

**Table 5:** GWAS summary for ThW and ThP traits

Traits	Chromosome	Positions (bp)/region (Mb)	SNP number	MAF	β^1)^	PVE^2)^	Candidate genes
ThW	1	55911047–56322618	71	0.10–0.16	7.88–10.95	4.56–5.60	*TBXAS1, HIPK2, ENSGALG00000012796*
ThW	4	45356293–45494740	6	0.06–0.07	11.24–15.37	4.63–6.85	*BMP3*
ThW	6	12624077	1	0.15	7.81	4.91	*ENSGALG00000030179*
ThW	7	1925186	1	0.05	13.6	4.88	*ZNF804A*
ThW	13	18054505–18280258	7	0.26–0.34	6.43–6.83	4.54–4.98	*KCTD16, NR3C1, ARHGAP26*
ThW	15	9905165–9919308	6	0.15–0.38	−6.30–−8.01	4.67–5.43	*CIT*
ThW	27	6056724–6138664	33	0.32–0.50	−5.76–−6.51	4.56–5.92	*IGF2BP1, GIP, SNF8*, etc.
ThP	3	64231641–65839287	5	0.07–0.08	−0.45–−0.51	4.62–5.20	*ENSGALG00000015017*
ThP	4	89236078–89259035	3	0.32–0.34	−0.27–−0.29	4.55–4.65	*ATRN*
ThP	6	27658260	1	0.23	−0.33	4.84	\
ThP	8	23488943	1	0.47	0.25	4.88	*BEND5*

1) β indicates allele effect calculated from GWAS result.

2) PVE indicates the proportion of phenotypic variance explained by SNPs.

GWAS, genome-wide association studies; SNPs, single-nucleotide polymorphismst; ThW, thigh muscle weight; ThP, percentage of thigh muscle weight; MAF, minor allele frequency.

## SUPPLEMENTARY MATERIAL

**Supplement 1**. Specific primers for candidate genes for RT-PCR.

**Supplement 2**. Fine mapping of EP trait on GGA27.

**Supplement 3**. LD block result based on significant SNPs.

**Supplement 4**. Effect of candidate haplotype on EP trait.

**Supplement 5**. Effect of second candidate haplotype on ThW traits.

**Supplement 6**. Effect of third candidate haplotype on ThW traits.

**Supplement 7**. Fine mapping of ThW trait on GGA1.

**Supplement 8**. LD block result based on significant SNPs on GGA1.

**Supplement 9**. Effect of first candidate haplotype on ThW traits.

**Supplement 10**. Effect of second candidate haplotype on ThW traits.

**Supplement 11**. Effect of third candidate haplotype on ThW traits.

**Supplement 12**. LD block result based on significant SNPs on GGA4.

**Supplement 13**. Effect of candidate haplotype on ThW traits.

**Supplement 14**. Fine mapping of ThW trait on GGA4.

**Supplement 15**. Annotation results of 73 candidate genes from GWAS summary of six carcass traits.
