# Supplementary material for: Estimation of genetic parameters and genome-wide association study for carcass traits in native chickens
Source: Anim Biosci. 2025 Apr 4;38(7):1328–41. doi: 10.5713/ab.25.0070 (PMC12229932; doi:10.5713/ab.25.0070)
Supplement: Supplementary file 1 [file ab-25-0070-Supplementary-1.pdf]

Supplement 1. Specific primers for candidate genes for RT-PCR.

Gene		Primer	Product length (bp)
IGF2BP1	F	AAGGCACAAGGCAGGATT	148
	R	GCAGCTCATTGACGGTTTT	
BMP3	F	GACAAGGTGTCGGAGCACA	160
	R	CATACAGCTCCTGGCTTCCA	
RASGEF1B	F	CTGGCAGATGGCCTGGATAG	280
	R	TCTGTGGCATCCTTTCCTGT	
ACSL5	F	CCCTAAAGGTGCCATGCTGA	170
	R	CTCCGCAGCTGTACATCACA	
CCND2	F	CCCGGAATGAAGAACTCTCCC	125
	R	TCAGGTACTTTCCACGCCAC	
GIP	F	GATGCACAGACGCTACTCGG	153
	R	GGCTCGGCTTCTCTCTTGTA	
MRPL22	F	GGCCATGCTTTACAGTCCCT	114
	R	TTACAGCCGAAGACGTGGAC	
ABI3	F	TCCCTCAGAGCTACCTGGAC	234
	R	ACTGTGTGGAACTCCTGGAC	
β-actin	F	GAGAAATTGTGCGTGACATCA	152
	R	CCTGAACCTCTCATTGCCA	
