# Supplementary figures and images for: Estimation of genetic parameters and genome-wide association study for carcass traits in native chickens
Source: Anim Biosci. 2025 Apr 4;38(7):1328–41. doi: 10.5713/ab.25.0070 (PMC12229932; doi:10.5713/ab.25.0070)

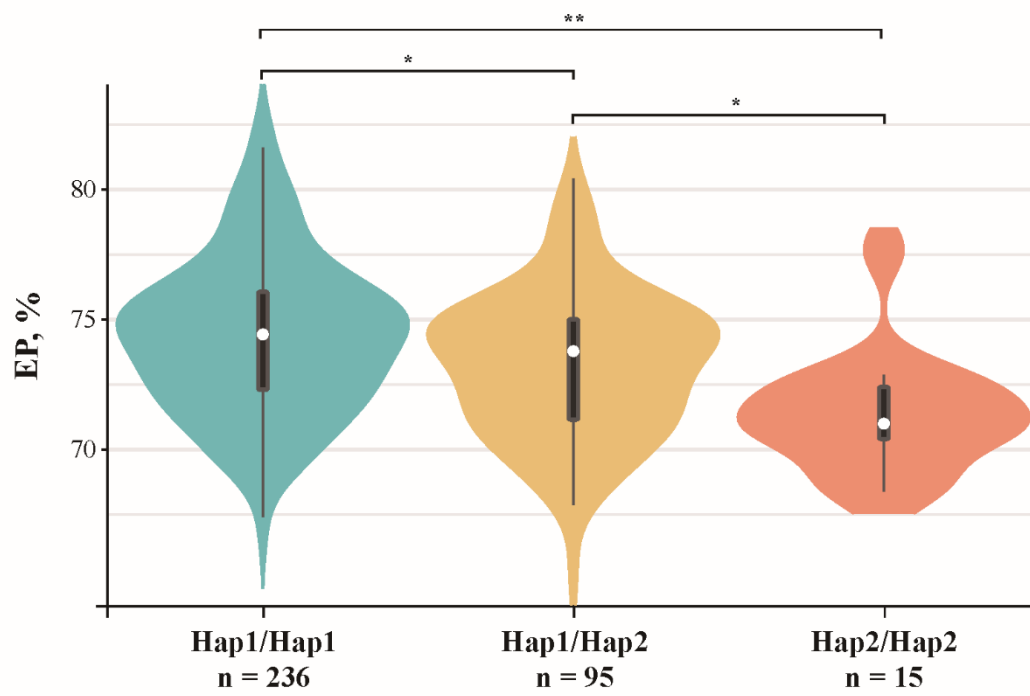

**Supplement 4.** Effect of candidate haplotype on EP trait. \* indicates  $p < 0.05$ , \*\* indicates  $p < 0.01$ .

Supplement: Supplementary file 4 [file ab-25-0070-Supplementary-4.pdf]

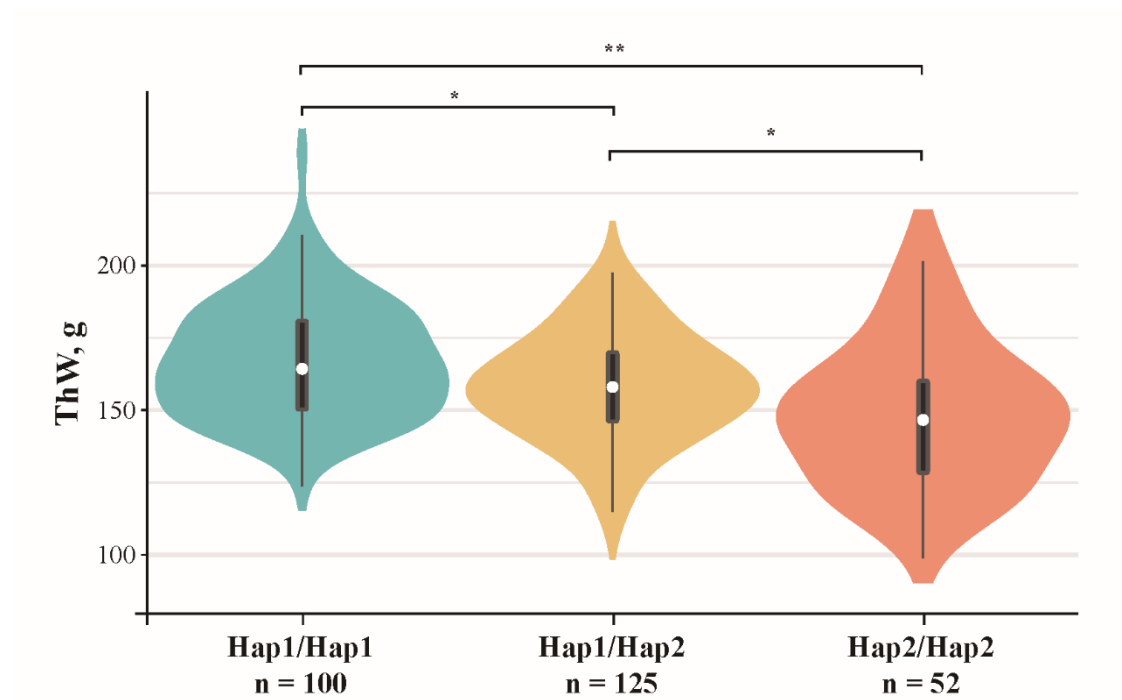

**Supplement 5.** Effect of second candidate haplotype on ThW traits. \* indicates  $p < 0.05$ , \*\* indicates  $p < 0.01$ .

Supplement: Supplementary file 5 [file ab-25-0070-Supplementary-5.pdf]

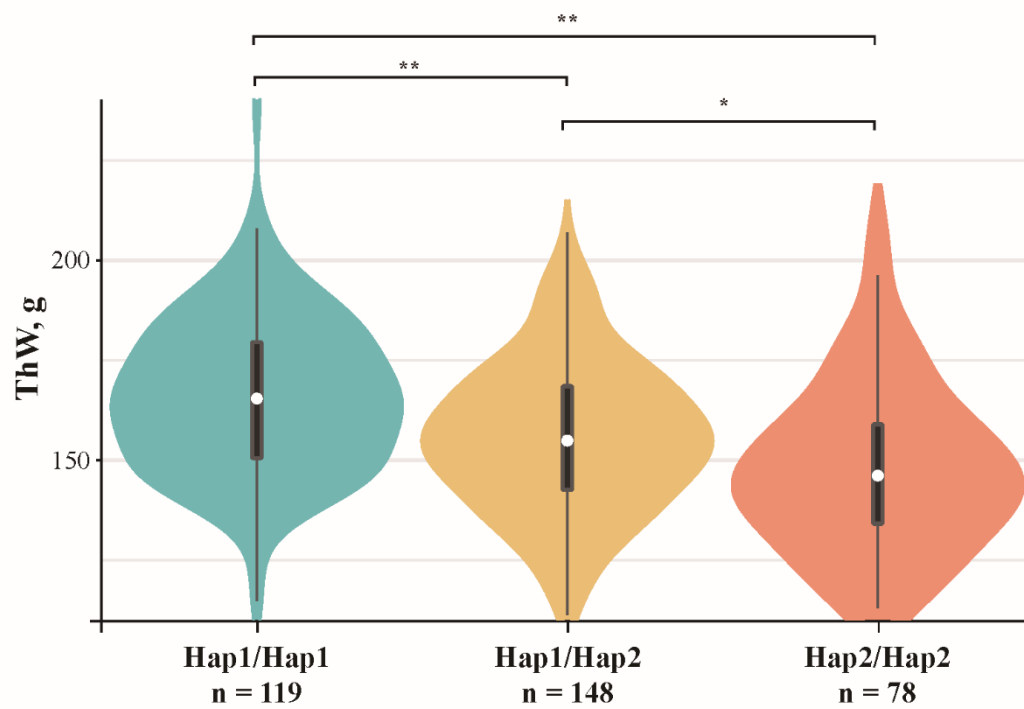

**Supplement 6.** Effect of third candidate haplotype on ThW traits. \* indicates  $p < 0.05$ , \*\* indicates  $p < 0.01$ .

Supplement: Supplementary file 6 [file ab-25-0070-Supplementary-6.pdf]
