# Supplementary material for: Estimation of genetic parameters and genome-wide association study for carcass traits in native chickens
Source: Anim Biosci. 2025 Apr 4;38(7):1328–41. doi: 10.5713/ab.25.0070 (PMC12229932; doi:10.5713/ab.25.0070)
Supplement: Supplementary file 9 [file ab-25-0070-Supplementary-9.pdf]

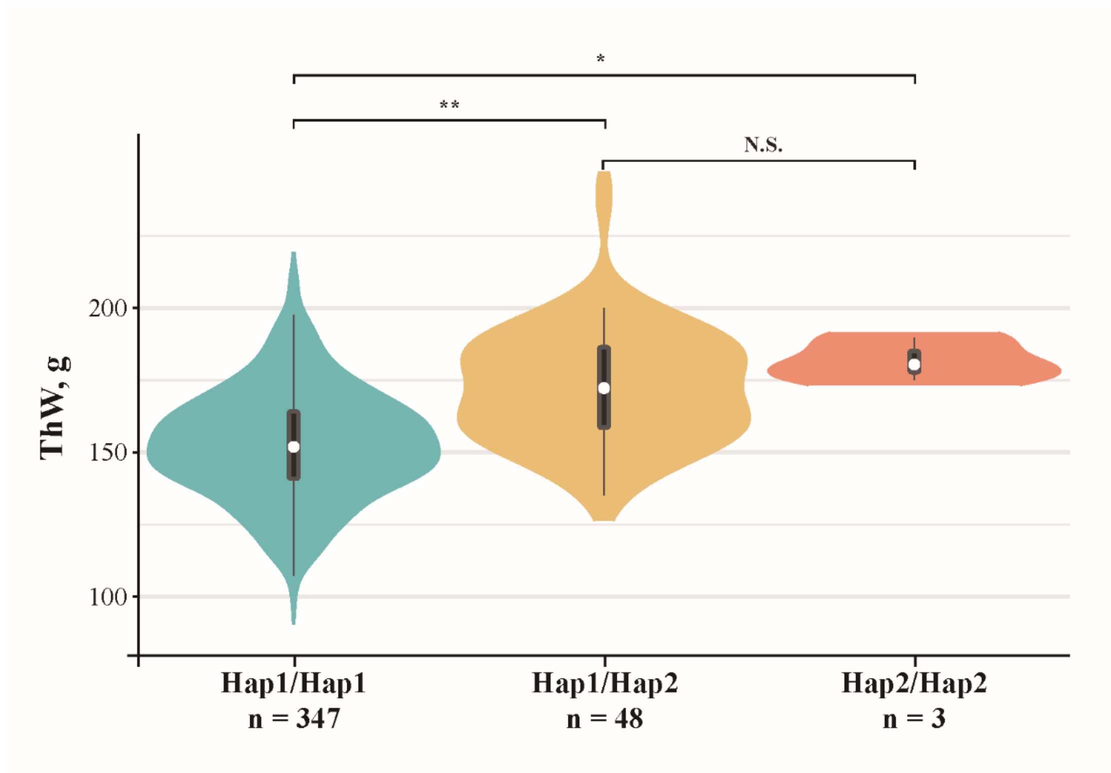

Supplement 9. Effect of first candidate haplotype on ThW traits. * indicates $p < 0.05$, ** indicates $p < 0.01$, N.S. indicates no significance.
