# Supplementary material for: Estimation of genetic parameters and genome-wide association study for carcass traits in native chickens
Source: Anim Biosci. 2025 Apr 4;38(7):1328–41. doi: 10.5713/ab.25.0070 (PMC12229932; doi:10.5713/ab.25.0070)
Supplement: Supplementary file 10 [file ab-25-0070-Supplementary-10.pdf]

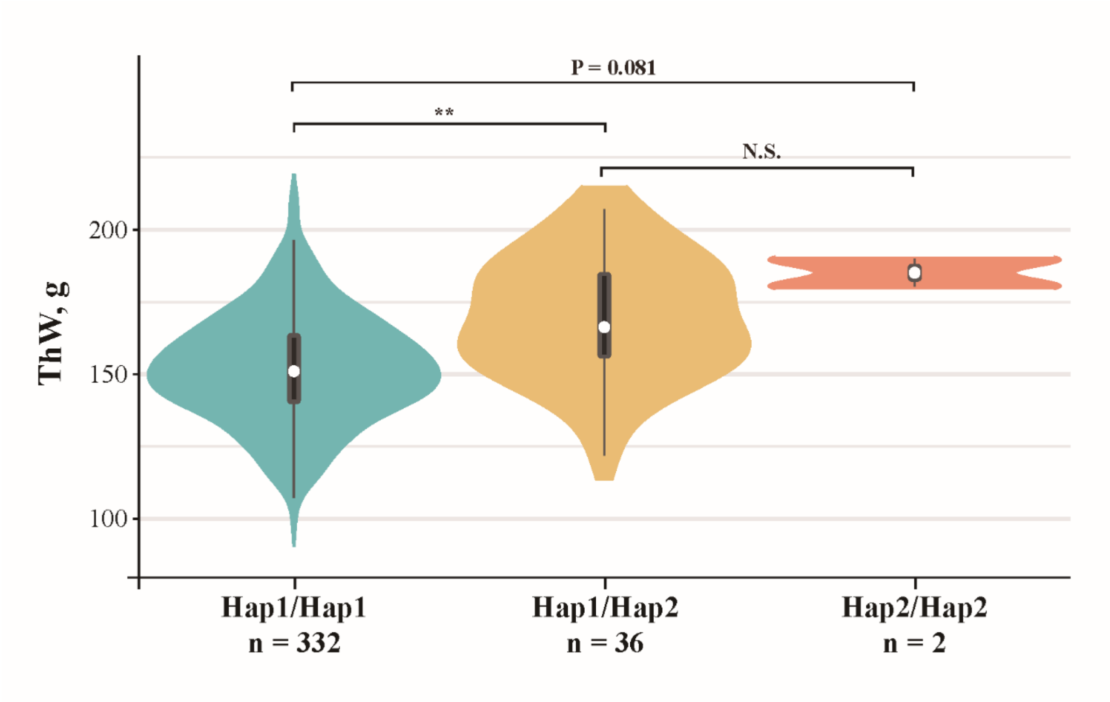

Supplement 10. Effect of second candidate haplotype on ThW traits. ** indicates p < 0.01, N.S. indicates no significance.
