# Supplementary material for: Estimation of genetic parameters and genome-wide association study for carcass traits in native chickens
Source: Anim Biosci. 2025 Apr 4;38(7):1328–41. doi: 10.5713/ab.25.0070 (PMC12229932; doi:10.5713/ab.25.0070)
Supplement: Supplementary file 15 [file ab-25-0070-Supplementary-15.pdf]

Supplement 15. Annotation results of 73 candidate genes from GWAS summary of six carcass traits.

ID	Gene name	Chr	Gene start (bp)	Gene end (bp)	Strand	Gene description
ENSGALG0000004407	*ABI3*	27	6041139	6044622	-1	ABI family member 3 [Source:NCBI gene;Acc:419988]
ENSGALG0000000884	*ACSL5*	6	27661391	27681605	1	acyl-CoA synthetase long-chain family member 5 [Source:NCBI
ENSGALG0000001307	*AKR1B10*	1	62458428	62466587	-1	aldo-keto reductase family 1 member B10 [Source:NCBI gene;Acc:395338]
ENSGALG0000005385	*AKR1E2*	1	62127588	62136095	-1	aldo-keto reductase family 1 member E2 [Source:NCBI gene;Acc:418171]
ENSGALG0000001727	*ANO2*	1	74343324	74478720	-1	anoctamin 2 [Source:HGNC Symbol;Acc:HGNC:1183]
ENSGALG0000003393	*ARHGAP26*	13	17989659	18090319	1	Rho GTPase activating protein 26 [Source:NCBI gene;Acc:396113]
ENSGALG0000001603	*ATRN*	4	89176735	89316390	1	attractin [Source:NCBI gene;Acc:422946]
ENSGALG0000001560	*BACH2*	3	75307454	75404609	1	BTB domain and CNC homolog 2 [Source:NCBI gene;Acc:421809]
ENSGALG0000003690	*BEND5*	8	23047528	23885562	-1	ATP/GTP binding protein like 4 [Source:NCBI gene;Acc:772083]
ENSGALG0000003505	*BMP3*	4	45457337	45471666	1	bone morphogenetic protein 3 [Source:NCBI gene;Acc:422581]
ENSGALG0000001278	*BMP6*	2	64378425	64463337	-1	bone morphogenetic protein 6 [Source:NCBI gene;Acc:420868]
ENSGALG0000001728	*C12orf4*	1	73358186	73385073	1	chromosome 1 open reading frame, human C12orf4 [Source:NCBI
ENSGALG0000003347	*CALD1*	1	62359691	62455538	1	caldesmon 1 [Source:NCBI gene;Acc:373965]
ENSGALG0000001728	*CCND2*	1	73471157	73515295	-1	cyclin D2 [Source:NCBI gene;Acc:374047]
ENSGALG0000003682	*CDH23*	6	12523595	12618142	1	cadherin related 23 [Source:NCBI gene;Acc:423718]
ENSGALG0000000735	*CIT*	15	9851336	9920684	1	citron rho-interacting serine/threonine kinase [Source:NCBI
ENSGALG0000003849	*CMIP*	11	15556046	15651941	1	c-Maf inducing protein [Source:NCBI gene;Acc:425774]
ENSGALG0000000208	*DOCK2*	13	4712213	4872139	-1	dedicator of cytokinesis 2 [Source:NCBI gene;Acc:427612]
ENSGALG0000001279	*DSP*	2	64524991	64561164	-1	desmoplakin [Source:NCBI gene;Acc:420869]

ENSGALG0000001622	*EFHC2*	1	112372336	1.12E+08	1	EF-hand domain containing 2 [Source:NCBI gene;Acc:418559]
ENSGALG0000001729	*FAR2*	1	73191857	73320608	1	fatty acyl-CoA reductase 2 [Source:NCBI gene;Acc:419043]
ENSGALG0000002779	*FGF23*	1	73425405	73429018	1	fibroblast growth factor 23 [Source:NCBI gene;Acc:428104]
ENSGALG0000001432	*FOXJ2*	1	75476115	75504709	-1	forkhead box J3 [Source:NCBI gene;Acc:418259]
ENSGALG0000000129	*GIP*	27	6090482	6098133	1	gastric inhibitory polypeptide [Source:NCBI gene;Acc:419989]
ENSGALG0000003120	*GNGT2*	27	6045993	6048245	1	G protein subunit gamma transducin 2 [Source:NCBI gene;Acc:100859179]
ENSGALG0000001279	*HIPK2*	1	56021548	56144368	1	homeodomain interacting protein kinase 2 [Source:NCBI gene;Acc:374138]
ENSGALG0000003315	*HOXB13*	27	6151929	6153522	1	homeobox B13 [Source:NCBI gene;Acc:771676]
ENSGALG0000005086	*HSD3B1*	1	79311694	79327367	-1	hydroxy-delta-5-steroid dehydrogenase, 3 beta- and steroid delta-isomerase 1
ENSGALG0000004120	*IGF2BP1*	27	6056993	6081137	-1	insulin like growth factor 2 mRNA binding protein 1 [Source:NCBI
ENSGALG0000001445	*ING4*	1	77734990	77749769	-1	inhibitor of growth family member 4 [Source:NCBI gene;Acc:418281]
ENSGALG0000001232	*KCTD16*	13	18270817	18306102	1	potassium channel tetramerization domain containing 16 [Source:NCBI
ENSGALG0000001442	*KPNA1*	1	77477877	77514773	-1	karyopherin subunit alpha 1 [Source:NCBI gene;Acc:418271]
ENSGALG0000001014	*MDGA1*	3	30006735	30187312	1	MAM domain containing glycosylphosphatidylinositol anchor 1 [Source:NCBI
ENSGALG0000004732	*MFAP5*	1	75647640	75660701	-1	microfibrillar associated protein 5 [Source:NCBI gene;Acc:418256]
ENSGALG0000001304	*MICAL3*	1	61867663	61967208	-1	microtubule associated monooxygenase, calponin and LIM domain containing 3
ENSGALG0000001551	*MMS22L*	3	72435233	72521377	1	MMS22 like, DNA repair protein [Source:NCBI gene;Acc:421799]
ENSGALG0000000391	*MRPL22*	13	12712730	12720801	-1	mitochondrial ribosomal protein L22 [Source:NCBI gene;Acc:416253]
ENSGALG0000001728	*NDUFA9*	1	73689598	73711387	1	NADH:ubiquinone oxidoreductase subunit A9 [Source:NCBI
ENSGALG0000003765	*NECAP1*	1	75447599	75455190	-1	NECAP endocytosis associated 1 [Source:NCBI gene;Acc:770083]

ENSGALG0000003299	*NR3C1*	13	18099574	18198040	-1	nuclear receptor subfamily 3 group C member 1 [Source:NCBI
ENSGALG0000005352	*NTF3*	1	74215223	74288369	1	neurotrophin 3 [Source:NCBI gene;Acc:428099]
ENSGALG0000001426	*PHC1*	1	75865514	75884654	1	polyhomeotic homolog 1 [Source:NCBI gene;Acc:418252]
ENSGALG0000003368	*PHOSPHO1*	27	6034509	6040276	1	phosphoethanolamine/phosphocholine phosphatase [Source:NCBI
ENSGALG0000001548	*PNISR*	3	71486522	71511444	1	PNN interacting serine and arginine rich protein [Source:NCBI
ENSGALG0000003128	*PTPRM*	2	98932396	99391529	-1	protein tyrosine phosphatase, receptor type M [Source:NCBI gene;Acc:421049]
ENSGALG0000001090	*RASGEF1B*	4	45493922	45604213	1	RasGEF domain family member 1B [Source:NCBI gene;Acc:100859744]
ENSGALG0000004018	*RIMKLB*	1	75676381	75723185	1	ribosomal modification protein rimK like family member B [Source:NCBI
ENSGALG0000001701	*RNASEH2B*	1	171220977	1.71E+08	1	ribonuclease H2 subunit B [Source:NCBI gene;Acc:418874]
ENSGALG0000000388	*SGCD*	13	12229467	12420212	-1	sarcoglycan delta [Source:NCBI gene;Acc:416251]
ENSGALG0000002730	*SKAP1*	27	6335894	6459922	1	src kinase associated phosphoprotein 1 [Source:NCBI
ENSGALG0000000561	*SLC44A3*	8	13840231	13932290	-1	solute carrier family 44 member 3 [Source:NCBI gene;Acc:424486]
ENSGALG0000000130	*SNF8*	27	6094182	6101707	1	SNF8, ESCRT-II complex subunit [Source:NCBI gene;Acc:419990]
ENSGALG0000000194	*SPDL1*	13	4874945	4897890	-1	spindle apparatus coiled-coil protein 1 [Source:NCBI gene;Acc:416172]
ENSGALG0000001279	*TBXAS1*	1	55750244	55990181	-1	thromboxane A synthase 1 [Source:NCBI gene;Acc:418101]
ENSGALG0000001434	*TEAD4*	1	75219390	75286457	-1	TEA domain transcription factor 4 [Source:NCBI gene;Acc:395542]
ENSGALG0000003469	*TMEM52B*	1	79164856	79169339	-1	transmembrane protein 52B [Source:NCBI gene;Acc:426455]
ENSGALG0000001342	*TULP3*	1	75291781	75327340	-1	tubby like protein 3 [Source:NCBI gene;Acc:426085]
ENSGALG0000000131	*UBE2Z*	27	6104309	6113729	-1	ubiquitin conjugating enzyme E2 Z [Source:NCBI gene;Acc:419991]
ENSGALG0000001547	*USP45*	3	71429787	71482168	1	ubiquitin specific peptidase 45 [Source:NCBI gene;Acc:421793]

ENSGALG0000001727	*VWF*	1	74541146	74683073	-1	von Willebrand factor [Source:NCBI gene;Acc:419031]
ENSGALG0000003259	*ZNF384*	1	77755770	77781779	-1	zinc finger protein 384 [Source:NCBI gene;Acc:770350]
ENSGALG0000000127	*ZNF652*	27	6001003	6022400	1	zinc finger protein 652 [Source:NCBI gene;Acc:419987]
ENSGALG0000002388	*ZNF804A*	7	1828066	1959021	-1	zinc finger protein 804A [Source:NCBI gene;Acc:423994]
ENSGALG00000030179		6	12620516	12626469	-1	chromosome 6 C10orf54 homolog [Source:NCBI gene;Acc:769500]
ENSGALG00000026776		1	73443329	73462784	-1	TP53 induced glycolysis regulatory phosphatase [Source:NCBI
ENSGALG00000032836		1	112544502	1.13E+08	1	monoamine oxidase B [Source:NCBI gene;Acc:418561]
ENSGALG00000048815		1	75938496	75943880	-1	
ENSGALG00000001525		27	6118078	6127639	1	calcium binding and coiled-coil domain 2 [Source:NCBI gene;Acc:419993]
ENSGALG00000012796		1	56172463	56308924	1	KIAA1549 [Source:NCBI gene;Acc:100857885]
ENSGALG00000015017		3	65815544	65827995	1	tubulin epsilon 1 [Source:NCBI gene;Acc:421750]
ENSGALG00000041372		2	43943919	43986021	-1	
ENSGALG00000041564		1	77147561	77281468	-1	mannosidase alpha class 1A member 2 [Source:NCBI gene;Acc:418265]
ENSGALG00000053279		3	65832266	65837554	-1	
